# Clinical use of computational modeling for surgical planning of arteriovenous fistula for hemodialysis

**DOI:** 10.1186/s12911-017-0420-x

**Published:** 2017-03-14

**Authors:** Michela Bozzetto, Stefano Rota, Valentina Vigo, Francesco Casucci, Carlo Lomonte, Walter Morale, Massimo Senatore, Luigi Tazza, Massimo Lodi, Giuseppe Remuzzi, Andrea Remuzzi

**Affiliations:** 1 Department of Biomedical Engineering, IRCCS-Istituto di Ricerche Farmacologiche “Mario Negri”, Bergamo, Italy; 2 Unit of Nephrology and Dialysis, ASST Papa Giovanni XXIII, Bergamo, Italy; 3 Unit of Nephrology and Dialysis, Ente Ecclesiastico “F. Miulli”, Acquaviva delle Fonti, BA Italy; 4 Unit of Nephrology and Dialysis, A.O. Cannizzaro, Catania, Italy; 5 Unit of Nephrology and Dialysis, Ospedale Annunziata, Cosenza, Italy; 6 Department of Urology and Nephrology, Catholic University, Rome, Italy; 7 Unit of Nephrology and Dialysis, Ospedale S. Spirito, Pescara, Italy; 8 Department of Biomedical and Clinical Sciences, University of Milan, Milan, Italy; 9 Department of Management, Information and Production Engineering, University of Bergamo, Bergamo, Italy

**Keywords:** Arteriovenous fistula, Computational modeling, Surgical planning, Hemodialysis vascular access

## Abstract

**Background:**

Autogenous arteriovenous fistula (AVF) is the best vascular access (VA) for hemodialysis, but its creation is still a critical procedure. Physical examination, vascular mapping and doppler ultrasound (DUS) evaluation are recommended for AVF planning, but they can not provide direct indication on AVF outcome. We recently developed and validated in a clinical trial a patient-specific computational model to predict pre-operatively the blood flow volume (BFV) in AVF for different surgical configuration on the basis of demographic, clinical and DUS data. In the present investigation we tested power of prediction and usability of the computational model in routine clinical setting.

**Methods:**

We developed a web-based system (AVF.SIM) that integrates the computational model in a single procedure, including data collection and transfer, simulation management and data storage. A usability test on observational data was designed to compare predicted vs. measured BFV and evaluate the acceptance of the system in the clinical setting. Six Italian nephrology units were involved in the evaluation for a 6-month period that included all incident dialysis patients with indication for AVF surgery.

**Results:**

Out of the 74 patients, complete data from 60 patients were included in the final dataset. Predicted brachial BFV at 40 days after surgery showed a good correlation with measured values (in average 787 ± 306 vs. 751 ± 267 mL/min, *R* = 0.81, *p* < 0.001). For distal AVFs the mean difference (±SD) between predicted vs. measured BFV was −2.0 ± 20.9%, with 50% of predicted values in the range of 86–121% of measured BFV. Feedbacks provided by clinicians indicate that AVF.SIM is easy to use and well accepted in clinical routine, with limited additional workload.

**Conclusions:**

Clinical use of computational modeling for AVF surgical planning can help the surgeon to select the best surgical strategy, reducing AVF early failures and complications. This approach allows individualization of VA care, with the aim to reduce the costs associated with VA dysfunction, and to improve AVF clinical outcome.

**Electronic supplementary material:**

The online version of this article (doi:10.1186/s12911-017-0420-x) contains supplementary material, which is available to authorized users.

## Background

Autogenous arteriovenous fistula (AVF) is the best choice for providing an efficient and long-lasting vascular access (VA) for hemodialysis (HD) patients, but it still has low primary and secondary patency rate [1]. The goal of AVF surgery is to obtain a blood flow volume (BFV) that allows a flow rate of at least 300 mL/min of blood within the HD extracorporeal circulation. On the other hand very high BFV, exceeding 1.5 L/min, should be avoided, for the risk for cardiac dysfunction and hand ischemia [2]. Vascular access dysfunction and complications such as non-maturation, failure, hand ischemia and risk of heart failure are important open clinical challenges [3]. There is a general consensus that careful planning of VA in end-stage renal disease patients approaching renal replacement therapy by HD is of crucial importance. Thus, patient physical examination, vascular mapping and Doppler ultrasound (DUS) evaluation of vessels are recommended for AVF planning [4, 5]. These procedures can suggest if patient vasculature structure and function are adequate for creation of a native fistula or if potential problems may develop during or after AVF surgery. However, they cannot provide an indication to the surgeon on the real outcome of the planned anastomosis in terms of AVF blood flow that will be obtained after the process of vessel remodeling and the consequent maturation of the VA. An objective and reliable prediction of post-operative BFV over time could be extremely important for planning the optimal AVF configuration. This information, if available, could guide the surgeon in the choice of location and type of anastomosis. Actually, it would be useful to know pre-operatively if BFV is predicted to be too low or too high, suggesting the risk of non-maturation of VA or risk for cardiac failure, respectively.

We have previously reported that a computational model [6], inserted in an open-source numerical solver [7], for prediction of AVF outcome, was successfully used to simulate clinical data obtained by expert clinical centers during a controlled clinical trial [8, 9]. The result of the computation allows to predict the effect of an AVF anastomosis type (end-to-side, end-to-end or side-to-side) and location (upper, middle or lower arm) in terms of changes in BFV, arterial and venous diameters that develop during AVF maturation, the process in which the artery and the vein importantly remodel to accommodate the increase in BFV resulting from direct connection of the arterial circulation to the vein. The tool has been validated in a prospective, observational and multicenter clinical study [9]. On the basis of these results the aim of our investigation was to develop and validate a system that allows using the computational tool in clinical practice.

The use of new technology in the clinical setting, and especially for computational modeling, needs careful evaluation of their effective usability and potential benefit in this critical environment. There are some important aspects that have to be considered in this regard. Firstly, clinical routine data generated may be not as accurate as those generated within a controlled clinical study. Secondly, a new tool should not increase the workload of the clinicians that is lately continuing increasing. With these constrains, we developed a web-based system (AVF.SIM) for simple and easy data transfer and fast and effective consultation of predicted results on planned AVF outcome. We then monitored six clinical centers during the use of the system in the routine clinical practice. We used observational pre- and post-operative clinical data to compare predicted BFVs with measured post-operative data. In addition, we evaluated the usability of the system on the basis of the user feedback.

## Methods

### The computational model

The computer code we used in our investigation is based on a pulse wave propagation network model (0D/1D) of the circulation previously developed [10, 11]. Briefly, in the vascular network segments and nodes are connected on the basis of main anatomical configuration. Each segment is modeled with an electric circuit using hydraulic analogy and model solution is based on the computation of hydraulic pressure and BFV for each element from conservation of mass and momentum, by assuming fully developed incompressible Newtonian blood flow in a straight tube. For each element three parameters are defined: hydraulic resistance and inductance per unit length, and vessel compliance, that incorporates the storage capacity of the segment. Changes in venous compliance, as a function of pressure, were derived from clinical data reported in literature [12]. Blood flow in side branches is taken into account using end-segments with linear resistance and a non-linear element is used for simulation of the hemodynamics in AVF anastomosis. As regards boundary conditions, cardiac output is assumed according to cardiac index [13], while extravascular pressure is assumed equal to 0 mmHg.

As previously described [13], patient-specific vascular networks are obtained on the basis of a generic vascular network model derived from literature data [14], adapting geometrical vessel diameters and lengths according to body weight, height, age and sex of individual patient. Blood vessel wall elasticity was also adapted according to demographic and clinical data (presence of hypertension and diabetes).

To estimate changes in vessel dimensions and BFV induced by AVF maturation, the solver embeds a vascular adaptation algorithm, previously described [6], based on the assumption that vessel dilation takes place upon changes in BFV to maintain physiologic value of the peak wall shear stress acting on vascular endothelial cells [15].

### The AVF.SIM system

**Fig. 1:**
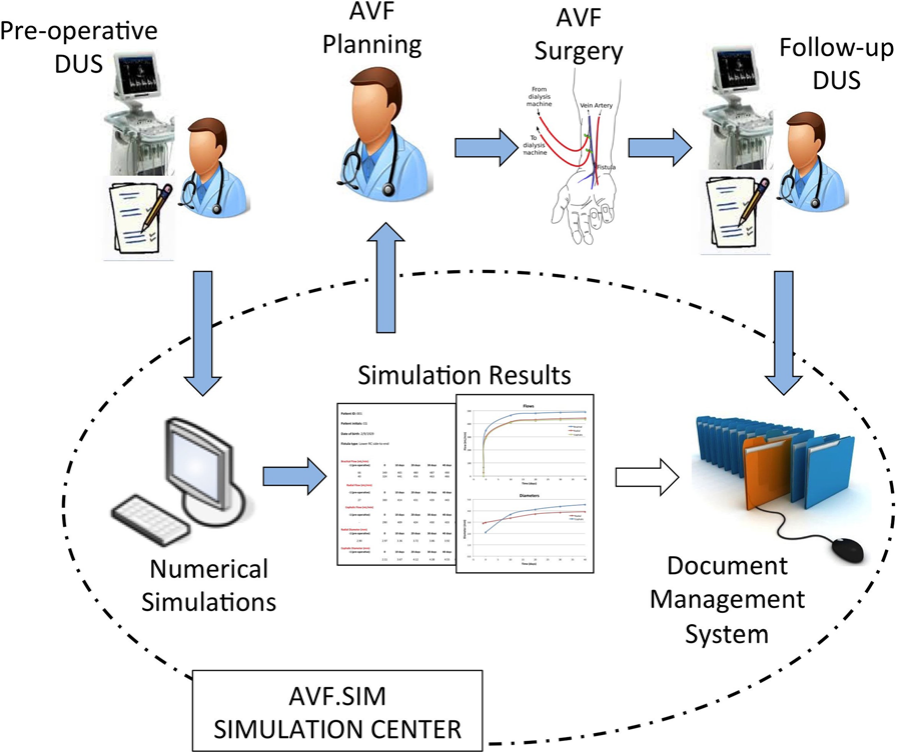
Representation of *AVF.SIM* system showing the procedure, that includes data collection and transfer, simulation management and data storing. Abbreviations: DUS, doppler ultrasound; AVF, arteriovenous-fistula

With the aim of introducing the computational model in the clinical setting, we developed the AVF.SIM system based on the flow of information represented in Fig. 1. As previously described [11], the first step of the procedure was the collection of demographic and clinical data in patients in need of AVF creation. Demographic data consist of patient age, gender, height and weight. Clinical data included haematocrit, plasma protein concentration, presence or absence of diabetes and/or hypertension. Subsequently, pre-operative DUS examination data were collected, including arterial and venous measurements according to the protocol described in detail below. Demographic information, clinical data and DUS measurements were collected by anonymized fillable .pdf form, where the nephrologist or the vascular surgeon also specifies the type and location of AVF they selected for surgery.

These pre-operative and anonymized forms were then sent by email to the Simulation Centre (Mario Negri Institute) and used for computer-based simulations. The results of the simulation were obtained in approximately half an hour. An output report (.pdf format) was then generated with computed time changes of BFV and diameters of arteries and veins (in selected locations) from the pre-operative condition up to 40 days after surgery, the typical time period in which AVF maturation takes place (see Additional file 1). These reports were then transmitted back to the clinicians by email and also made available through a secured web-based document management system (DMS), a repository for quick visualization of files protected by personal login. The users have restricted access to data of their own clinical centre only.

### Usability test of the AVF.SIM system

To evaluate the acceptance of AVF.SIM system in the clinical setting, a usability test with duration of 6 months was designed. Data were collected on the basis of routine clinical management of patients in need of VA to start renal replacement therapy by HD. Data on all incident dialysis patients selected for a native AVF in the observation period were considered, whereas data from patients designated for central venous catheter or synthetic arteriovenous graft were excluded from the present test. The following six centres took part to the test: *ASST-Papa Giovanni XXIII* (Bergamo), *Ospedale F. Miulli* (Acquaviva delle Fonti, Bari), *Ospedale Civile S. Spirito* (Pescara), *Azienda Ospedaliera Cannizzaro* (Catania), *Azienda Ospedaliera di Cosenza-Annunziata* (Cosenza), *Policlinico Universitario A. Gemelli* (Rome). The local Ethics Committee of coordinating center approved the study protocol. Consent for the use of clinical data was obtained from each patient before AVF surgery. Clinical data have been derived from routine patient records and no intervention was based on data generated during the usability test. Ten interventional nephrologists with several years of expertise in VA management, including pre-operative mapping, surgery and surveillance, were introduced to the system sharing the vascular protocol and instructions for data collection and transmission. All the nephrologists belong to the Vascular Access Working Group of the Italian Society of Nephrology.

**Fig. 2:**
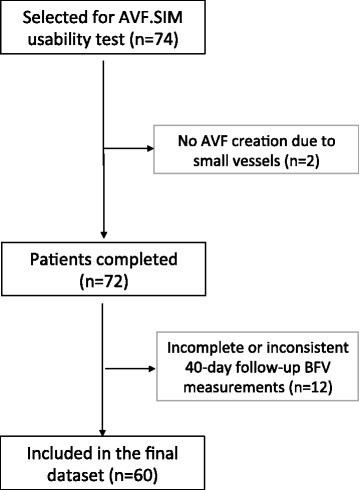
Flow diagram showing the number of patients selected for *AVF.SIM* usability test and the number of them included in the final dataset

As shown in Fig. 2, 74 patients were selected for enrolment in the study. All patients underwent clinical and vascular DUS examinations pre-operatively. For two patients AVF was not created due to very small blood vessel diameter. In 11 patients, AVF was created in the upper arm and was brachio-cephalic (BC). In 61 patients AVF was created in the lower arm and it was radio-cephalic (RC). The type of anastomosis was *side-to-end* (7 BC and 39 RC), *end-to-end* (14 RC) or *side-to-side* (9 RC and 3 BC).

For the comparison of computational prediction and the effective outcome of AVF surgery, a follow-up DUS examination was performed 40 days after surgery. Follow-up data on access BVF were inserted in an anonymized fillable.pdf form, transferred to the Simulation Centre using the same procedure used for pre-operative data. Out of the 72 patients, 40-day follow-up data were not provided in only one patient. Eleven patients were excluded from this analysis due to incomplete or inconsistent follow-up BFV measurements, mainly for brachial artery BFV lower than the sum of radial and ulnar artery BFV (Fig. 2). Demographic and clinical data, used to generate patient-specific vascular network models of the patients included in the final dataset, are summarized in Table 1. At the end of AVF.SIM usability test, a questionnaire was submitted to all clinicians, to measure their satisfaction and to collect suggestions for further development of the system and the user interface (see Additional file 2). All users were asked to fill in a questionnaire with twelve statements, using a level of agreement between 1 and 5, corresponding to the following scale: 1 = strongly disagree, 2 = disagree, 3 = not agree nor disagree, 4 = agree, 5 = strongly agree. To measure the satisfaction level, for every statement the mean of the score was calculated.

**Table 1:** Demographic and clinical data

	RC S-E	RC E-E	RC S-S	BC S-E	BC S-S
*Number of AVF*	32	13	5	7	3
Age (*years*)	60 ± 16	62 ± 15	67 ± 15	57 ± 14	72 ± 11
Gender *(females)*	13 (41%)	0 (0%)	0 (0%)	6 (86%)	3 (100%)
AVF arm *(right)*	8 (25%)	1 (8%)	1 (20%)	1 (14%)	1 (33%)
Height *(cm)*	168 ± 11	170 ± 9	168 ± 3	166 ± 7	158 ± 3
Weight *(kg)*	75 ± 18	75 ± 12	72 ± 8	61 ± 9	68 ± 11
Systolic Pressure (*mmHg*)	136 ± 17	145 ± 19	124 ± 18	139 ± 13	133 ± 15
Diastolic Pressure (*mmHg*)	76 ± 9	82 ± 14	74 ± 17	80 ± 6	83 ± 6
Hematocrit (%)	33 ± 5	34 ± 3	34 ± 5	36 ± 4	39 ± 1
Protein plasma concentration (g/dl)	6.5 ± 0.7	6.8 ± 0.6	6.5 ± 0.5	6.8 ± 0.7	6.3 ± 0.4
Hypertension *(yes)*	25 (78%)	11 (85%)	5 (100%)	7 (100%)	5 (100%)
Diabetes *(yes)*	6 (18%)	1 (8%)	0 (0%)	0 (0%)	1 (33%)

Values are mean ± s.d. for continuous variables or frequency (percentage) for gender, arm, hypertension and diabetes

*Abbreviations*: *AVF* arterio-venous fistula, *BC* brachio-cephalic, *RC* radio-cephalic, *E-E* end-to-end, *S-E* side-to-end, *S-S* side-to-side

### Protocol for vascular DUS evaluations

During the pre-operative DUS examination patients were in a standardized supine position with the examined arm parallel to the body in a comfortable position for imaging. Both for arterial and for venous side, diameters were assessed using B-mode images of vessels in their transverse view and consisted of short- and long-axis diameter measurements. Venous assessment included cephalic vein from the wrist to the cephalic arch, the cubital vein at the elbow, the basilic vein from the elbow until it joins the brachial vein in the upper arm and the subclavian vein immediately before the bifurcation of the cephalic arch and basilic vein. These measurements of venous vessels were assessed making use of a proximal pressure-cuff for congestion. Arterial assessment included brachial artery at middle-arm and at the elbow, radial and ulnar arteries both in the middle and distal positions and subclavian artery. For the measurements of subclavian artery and subclavian vein the patient was asked to hold his breath. On brachial, radial and ulnar artery the time average velocity (TAV) was also assessed. The operator was required to trace three complete cardiac cycles on the Velocity/Time curve calculated by pulsed-wave Doppler. This was used to calculate the BFV with the assumptions and procedure previously described [9, 15]. Standardization of these examinations was performed according to model requirements. During the follow-up DUS examination at 40 days after surgery, diameter measurements of brachial artery, radial artery and cephalic vein were performed. During this examination TAV was also assessed in brachial and radial artery and used to calculate BFV with the same procedure used for pre-operative examination.

### Statistical analysis

Data are expressed as Mean ± SD. The correlation between predicted and measured brachial artery BFV was investigated using the linear regression analysis. The agreement between predicted and measured BFV in distal AVFs was analyzed using Bland-Altman plot.

## Results

Data from 60 patients with newly created AVF were divided into groups based on AVF configuration: lower arm RC S-E (*n* = 32), lower arm RC E-E (*n* = 13), lower arm RC S-S (*n* = 5), upper arm BC S-E (*n* = 7) and upper arm BC S-S (*n* = 3). In general, a good agreement was observed between predicted and measured brachial artery BFV at 40 days after surgery, with average values of 787 ± 306 vs. 751 ± 267 mL/min, respectively. As expected, regression analysis between predicted and measured values of brachial artery BFV showed a strong and statistically significant linear correlation in the whole final dataset (*R* = 0.81, *p* < 0.001).

**Fig. 3:**
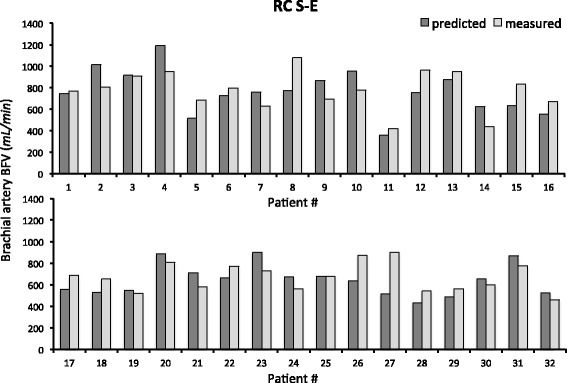
Comparison between *predicted* and *measured* brachial artery BFV at 40 days after AVF surgery in patients with *lower* arm *RC S-E AVF*. Abbreviations: BFV, blood flow volume; RC, radio-cephalic; S-E, side-to-end

**Fig. 4:**
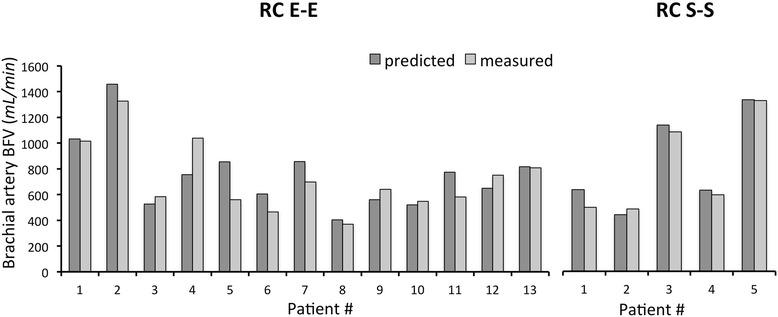
Comparison between *predicted* and *measured* brachial artery BFV at 40 days after AVF surgery in patients with *lower* arm *RC E-E* and *S-S AVF*. Abbreviations: BFV, blood flow volume; RC, radio-cephalic; E-E, end-to-end; S-S, side-to-side

In patients with newly created RC S-E AVF, the comparison between predicted and measured brachial artery BFV is reported in Fig. 3, showing a good accuracy of the simulations, with agreement between predicted and measured brachial BFV (704 ± 186 vs. 720 ± 166 mL/min, respectively), with very good prediction for some patients and some discrepancies in only two of them. Similarly, in RC E-E and S-S AVF, a good agreement was observed for E-E AVF between predicted and measured BFV (755 ± 274 vs. 722 ± 267 mL/min), and even a better prediction for BFV of S-S AVF (843 ± 383 vs. 804 ± 388 mL/min), as shown in Fig. 4.

**Fig. 5:**
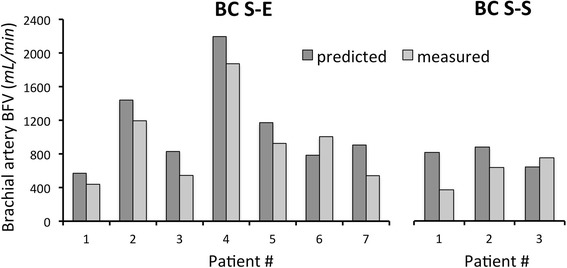
Comparison between *predicted* and *measured* brachial artery BFV at 40 days after AVF surgery in patients with *upper* arm *AVF*, *BC S-E*, *BC S-S* and *BB S-S AVF*. Abbreviations: BFV, blood flow volume; BC, brachio-cephalic; BB, brachio-basilic; S-E, side-to-end; S-S, side-to-side

**Fig. 6:**
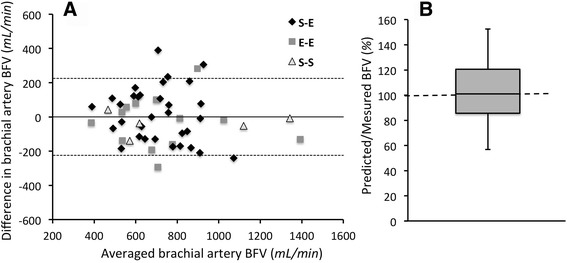
**a** *Bland-Altman plot* showing the agreement between *predicted* and *measured* brachial BFV at 40 days after AVF creation. Different *symbols denote* different AVF configurations. **b** *Box plot* showing the percent ratio between predicted and measured brachial artery BFV in distal AVFs. The *grey box* represents the second and third quartiles (range 86–121%, median 101%), the *above whisker* represents the maximum of data range (153%) and the *below whisker* the minimum (57%). Abbreviations: BFV, blood flow volume; E-E, end-to-end; S-E, side-to-end; S-S, side-to-side

We found less accurate agreement between predicted and measured results for proximal AVF (1065 ± 463 vs. 858 ± 438 mL/min), as shown in Fig. 5. Out of ten patients, important discrepancies were observed in two cases of S-E anastomosis and in one case of S-S configuration. For the limited number of proximal AVFs, further statistical analysis was conducted on results of distal AVFs only. The Bland-Altman plot, reported in Fig. 6a, shows a good accuracy between predicted and measured blood flow in distal AVF. The difference between predicted and measured BFV are uniformly distributed in the entire range of average brachial artery BFV (see Fig. 6a). The precision of the computed prediction is shown in Fig. 6b by the box-plot of relative percent difference between predicted and measured brachial BFV that averaged 1.98 ± 20.87%. For half of the simulations the percent difference was included in the range 86–121% (second and third quartiles).

The analysis of the data obtained through the questionnaires revealed that DUS vascular protocol was clear and exhaustive (mean score 4.6) and vascular measurements required by the protocol acceptable in the clinical routine (4.4). The procedure used for data collection and transmission was appropriate (4.7) and pre-operative and follow-up forms were useful for data collection (4.6). Results of simulations were provided with haste by the Simulation Center (4.2), the content of results files was clear and well organized (4.7) and was found to include all the data of interest (4.5). As regards data management, clinicians felt comfortable in receiving simulations results via email (4.6) and they appreciated to have the DMS as repository of all pre-operative data, simulations results and follow-up data (4.4). In conclusion, all the participants declared they were willing to use the AVF.SIM system for AVF planning (4.6).

## Discussion

In the present observational study we evaluated the power of prediction of AVF.SIM system and its acceptance in the routine clinical setting. This new system allowed the successful integration of the previously developed computational model in a single procedure that includes data collection and transfer, numerical simulation management and data storage. The good agreement between predicted and measured BFV at 40 days after surgery, both for distal and proximal AVF, confirmed that also in routine clinical settings the accuracy of prediction of the computational model is satisfactory, as it was during model validation obtained in a controlled clinical study. In particular, for distal AVFs the predicted values we obtained are in average very close to those measured (with an average difference of 1.98 ± 20.87% between predicted and measured BFV). Values of the difference between predicted and measured brachial BFV after maturation were within the range of 86–121% in 50% of patients, and in the range of 60–144% for 95% of cases.

As far as effective usability of the computational tool, the complete sets of data collected and the feedback provided by clinicians indicated that AVF.SIM system is easy to use and well accepted in clinical routine, with a limited additional workload for pre-operative examination by the clinicians. Actually the computed prediction of patient-specific BFVs after VA maturation are obtained using the AVF.SIM system on the basis of demographic information, clinical data and pre-operative DUS measurements, that are today all standard of care for patients in need of VA for HD treatment. Time required to fill up the pre-operative form and send data to the Simulation Center was estimated to extend the routine vascular mapping of approximately 10–15 min only. Pre-operative physical examination and DUS evaluation currently recommended by international guidelines [16] have several potential benefits, but only a system that takes into account the complex interplay of demographic and clinical factors, as well as vessel dimensions and local BFV, could really help the surgeon in identifying the best site for AVF placement, as well as in preventing very low or very high BFV, likely associated with VA complications. The establishment of effective usability of AVF.SIM in the clinical environment is a significant step forward allowing computer assisted clinical decision making on type and location of AVF, as well as for the evaluation of potential risks for VA non maturation. The development and validation of the computational model, despite important simplifications on vascular structure and function, allowed obtaining valuable patient-specific results, without the need of more invasive and complex procedures like pre-operative magnetic resonance imaging or angiography [17].

To date, few studies deal with prediction of AVF outcome. Lok et al. [18] presented a scoring system to stratify the patient’s risk for failure to mature, identifying in advanced age, peripheral vascular disease, coronary artery disease and white race the most significant clinical predictors. The Lok’s model, however, does not predict AVF outcome. In a recent study conducted by Masengu et al. [19], there is only general indication that female gender, lower arm and low radial artery BFV are associated with AVF failure. However, there is no general consensus on the criteria to select the type of anastomosis for AVF and the only suggestion is to avoid making AVF in vessels of small diameter [4], which means general indication for risk of failure, with no indication about the best AVF type and location for individual patients.

The use of AVF.SIM system in six clinical centers is the first attempt at introducing a predictive computational tool for AVF outcome in the clinical routine. The ability to predict blood flow and diameter may allow surgeons to identify the best site for placement of an AVF, that likely results in adequate BFV, guiding them in creating a different VA when the predicted flow at 40 days post operation is too low for all potential AVF sites, or suggesting a more distal site for AVF creation in case of predicted BFV very high for an upper-arm AVF. Thus, the clinical use of the present computational model potentially allows individualization of VA and a more patient-centered approach, advocated by nephrologists as the only strategy to succeed in VA creation [20, 21].

We have to acknowledge that accuracy of predicted BFV in proximal AVF is lower as compared with results obtained in distal AVFs. Comparisons between predicted and measured BFV in the brachial artery, that is upstream and far from the anastomosis site for distal AVFs, may minimize measurement inaccuracies related to the presence of disturbed flows [22]. In line with this observation, we can hypothesize that less accurate predictions of proximal AVF, as compared to distal AVFs, may be due to the fact that DUS examinations after VA maturation are more complex in proximal than in distal AVF, due to the proximity of the location for BFV measurement to the anastomosis. We also have to acknowledge that the number of patients excluded due to inconsistent or incomplete follow-up data is not negligible. Although to a lesser extent, the presence of disturbed flow at the anastomosis may affect the accuracy of BFV measurements also for distal AVF, resulting in radial BFV higher than brachial BFV. This may explain discrepancies we found in follow-up data and may justify our choice of excluding these patients from the final dataset. Another limitation of our investigation that may explain the inaccuracy of prediction in BFV after VA maturation, is that we assumed a constant 40 days time interval for AVF maturation for the whole patient population. While this assumption may be reasonable for most of cases, it could be erroneous for some of them, like diabetic patients, who have slower VA maturation [23]. Finally, the results of our usability test are also limited, in some way, by the small number of AVFs examined. However, the positive results we obtained suggest that a more extensive clinical evaluation of AVF.SIM is worth to perform. A larger, randomized and prospective study may provide more extensive evaluation of clinical efficacy of AVF.SIM, as well as on the potential improvement of AVF outcome as compared to conventional VA surgery planning.

## Conclusions

The clinical test of AVF.SIM system confirmed the accuracy of BFV predictions and established that the system is easy to use and well accepted in clinical routine. The use of this system to support selection of the optimal surgery procedure could help perform more efficient AVF planning, reducing non maturation events, late failure rate, and very high VA blood flows at risk for heart failure and steal syndrome. The use of the computational modeling, in addition to the currently performed DUS evaluations, may allow reduction of economic costs associated with VA dysfunction, as well as morbidity and mortality of HD patients.

## Additional files

Additional file 1: Example of report containing the results of simulation. (PDF 55 kb)
Additional file 2: Usability test questionnaire submitted to clinicians. (PDF 88 kb)

## Abbreviations

AVF: Arteriovenous fistula
BC: Brachio-cephalic
BFV: Blood flow volume
DMS: Document management system
DUS: Doppler Ultrasound
E-E: End-to-end
HD: Hemodialysis
RC: Radio-Cephalic
S-E: Side-to-end
S-S: Side-to-side
TAV: Time average velocity
VA: Vascular access
