# Supplementary material for: Clinical use of computational modeling for surgical planning of arteriovenous fistula for hemodialysis
Source: BMC Med Inform Decis Mak. 2017 Mar 14;17:26. doi: 10.1186/s12911-017-0420-x (PMC5348915; doi:10.1186/s12911-017-0420-x)
Supplement: Additional file 1: — Example of report containing the results of simulation. (PDF 55 kb) [file 12911_2017_420_MOESM1_ESM.pdf]

Patient ID: 014

Fistula type: Lower RC side-to-side

Brachial Flow (mL/min)

-1 (pre-operative)	0	10 days	20 days	30 days	40 days	
51	437	620	680	716	746	2A-BRACHIAL_MIDDLE
38	418	603	663	699	729	3A-BRACHIAL_LOWER

Radial Flow (mL/min)

-1 (pre-operative)	0	10 days	20 days	30 days	40 days	
22	418	603	663	699	729	5A-RADIAL DISTAL

Cephalic Flow (mL/min)

-1 (pre-operative)	0	10 days	20 days	30 days	40 days	
-	387	572	632	669	698	8V-CEPHALIC LOWER DIST

Radial Diameter (mm)

-1 (pre-operative)	0	10 days	20 days	30 days	40 days	
3.10	3.14	3.84	4.39	4.63	4.78	5A-RADIAL DISTAL

Cephalic Diameter (mm)

-1 (pre-operative)	0	10 days	20 days	30 days	40 days	
-	2.53	4.30	4.90	5.29	5.57	8V-CEPHALIC LOWER DIST

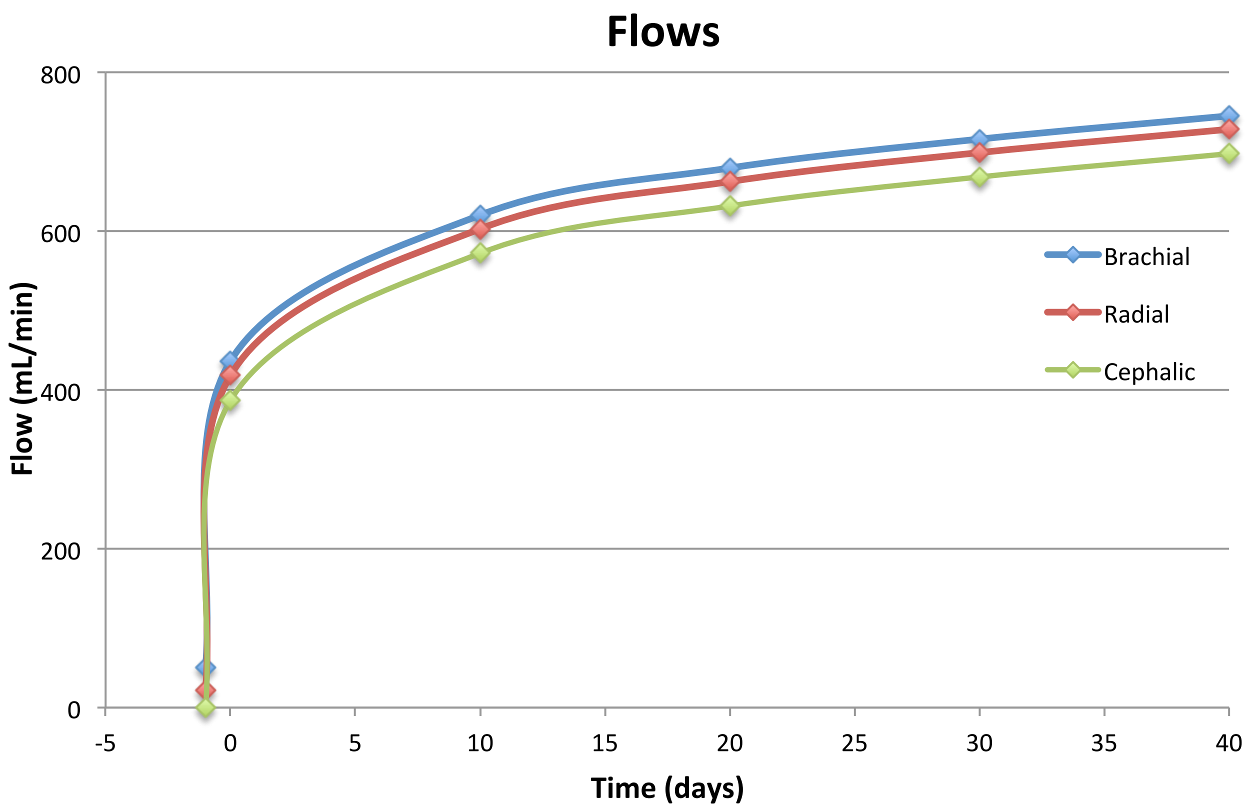
Flows
800
600
400
200
0
Flow (mL/min)
-5
0
5
10
15
20
25
30
35
40
Time (days)
Brachial
Radial
Cephalic

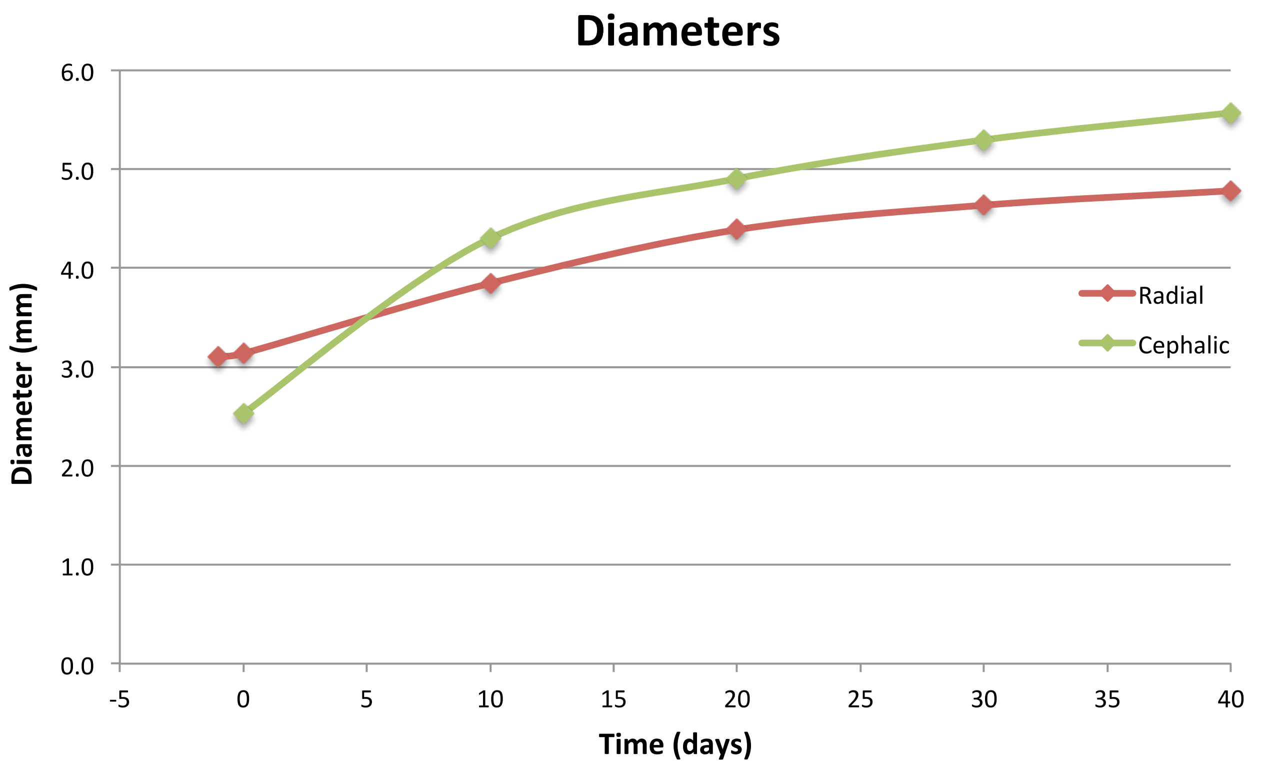
Diameters
6.0
5.0
4.0
3.0
2.0
1.0
0.0
Diameter (mm)
-5
0
5
10
15
20
25
30
35
40
Time (days)
Radial
Cephalic
