# Supplementary material for: Clinical use of computational modeling for surgical planning of arteriovenous fistula for hemodialysis
Source: BMC Med Inform Decis Mak. 2017 Mar 14;17:26. doi: 10.1186/s12911-017-0420-x (PMC5348915; doi:10.1186/s12911-017-0420-x)
Supplement: Additional file 2: — Usability test questionnaire submitted to clinicians. (PDF 88 kb) [file 12911_2017_420_MOESM2_ESM.pdf]

AVF.SIM SYSTEM QUESTIONNAIRE

At the end of AVF.SIM usability test, we will appreciate to receive your personal feedback about the system. Please fill this questionnaire and send it to the coordinator centre via email (avf.sim@marionegri.it) or fax (035-4535392). For every statement we kindly ask you to tick the appropriate box, that express your level of agreement in a range between 1 to 5, corresponding to the following scale:

Strongly Disagree	Disagree	Neither agree or disagree	Agree	Strongly Agree
1	2	3	4	5

Ultrasound vascular protocol

1. I found the US vascular protocol clear and exhaustive

1	2	3	4	5

2. I found vascular measurements required by the protocol acceptable in the clinical routine

1	2	3	4	5

Data collection and transmission

1. The fillable .pdf forms were appropriate for data collection

1	2	3	4	5

2. I experienced some difficulties in data transmission procedure

1	2	3	4	5

3. I found paper forms very useful for data collection

1	2	3	4	5

Simulations results

1. Results of simulations were provided with haste by the coordinator centre

1	2	3	4	5

2. Results files content was clear and well organized

☐ 1 ☐ 2 ☐ 3 ☐ 4 ☐ 5

3. Results files included all the data of interest

☐ 1 ☐ 2 ☐ 3 ☐ 4 ☐ 5

Data management – The web-based Document Management System (DMS)

1. I felt comfortable in receiving simulations results via email

☐ 1 ☐ 2 ☐ 3 ☐ 4 ☐ 5

2. I found useful to have the DMS as repository of all data

☐ 1 ☐ 2 ☐ 3 ☐ 4 ☐ 5

3. I found the DMS easy to access and its structures well integrated

☐ 1 ☐ 2 ☐ 3 ☐ 4 ☐ 5

In conclusion

I would like to use the AVF.SIM system in AVF planning

1	2	3	4	5
**Strongly Disagree**	**Disagree**	**Neither agree or disagree**	**Agree**	**Strongly Agree**

Any comments, suggestions, notes:

……

……
